# SARS-CoV-2 seroconversion in children attending daycare versus adults in Germany between October 2020 and June 2021

**DOI:** 10.1038/s43856-023-00352-3

**Published:** 2023-09-15

**Authors:** Ulrike Kubisch, Anna Sandoni, Juliane Wurm, Anja Schienkiewitz, Martin Schlaud, Tim Kuttig, Bianca Finkel, Susanne Jordan, Julika Loss

**Affiliations:** Department of Epidemiology and Health Monitoring, Robert Koch Institute, Berlin, Germany

**Keywords:** Viral infection, Epidemiology

## Abstract

**Background:**

Data on seroconversion rates after SARS-CoV-2 infection in young children (<6 years) is scarce. The present study compares seroconversion rates between young children and adults and identifies associated factors.

**Methods:**

The COALA study (“Corona-outbreak-related examinations in daycare centers”) investigated transmission dynamics of SARS-CoV-2 in daycare centers and associated households (10/2020-06/2021). 114 individuals tested positive for SARS-CoV-2 through PCR either prior to the study period by health authorities or in PCR testing during the study period. Two capillary blood samples were obtained within five weeks consecutively and tested for SARS-CoV-2 IgG-antibodies (second sampling depending on positive PCR). Results from 91 participants (38 young children 1–6 years, 53 adults) were included in the analyses.

**Results:**

Seroconversion rate in young children is significantly higher than in adults (97.4% versus 66%). High viral load and longer time interval between the probable date of infection and antibody testing are associated with seroconversion.

**Conclusions:**

Our findings depict substantial development of specific antibodies in young children after SARS-CoV-2 infection. This may provide temporary protection from re-infection for young children or severe disease for this age group.

## Plain language summary

When fighting an infectious disease, the immune system often produces antibodies. These proteins circulate in the blood, where they help to clear the infection and generally remain present for several months after recovery. Little is known about how often children younger than 6 years develop antibodies after SARS-CoV-2 infection. The aim of our study was to compare antibody development of young children and adults. We examined blood samples from young children and adults after SARS-CoV-2 outbreaks in daycare centers during the early pandemic (10/2020–06/2021) in Germany. Young children and adults who tested positive for SARS-CoV-2 had two blood samples taken at an interval of five weeks. We found that young children are more likely to develop antibodies after SARS-CoV-2 infection than adults. These findings indicate that young children may be—at least temporarily—protected from re-infection or from a severe course of the disease.

## Abstract (web summary)

Kubisch et al. examine antibody seroconversion rates after SARS-CoV-2 infection in children attending daycare centers and adults in Germany. They found higher seroconversion rates in children compared with adults, which may have an influence on reinfection severity in the younger population.

## Introduction

Seroconversion rate describes the proportion of individuals developing antibodies after antigen contact via an infection or vaccination in a given time period. Determining seroconversion rates after natural infection is important to assess immune protection and to develop and adjust vaccination strategies. Seroconversion after SARS-CoV-2 infection in adults has been described to occur as early as 7 days after symptom onset with seroconversion rates from 50% to 58.8%^1,2^ and up to 100% 14 days after symptom onset^2–4^. Studies concerning children, however, show a greater variety in their results regarding seroconversion duration and rates. For children and teenagers (0–18 years), seroconversion rates from 37% to 100% have been described, based on blood samples taken 14 days (and up to 172 days) after positive PCR testing or symptom onset^5–9^. This heterogeneity highlights the need for further investigation. For example, children under 6 years of age present an understudied, albeit relevant population group. Children under the age of 6 years are often enrolled in daycare programs, where SARS-CoV-2 transmission, due to close contact among young children, cannot be fully prevented. Vaccines for children were not approved in the European Union, and thus also in Germany, until November 2021 (5–11-year-olds) and October 2022 (6 months to 4-year-olds). At the same time, in both cases, the STIKO (Standing Committee on Vaccination) did not issue a general vaccination recommendation for the respective age groups^10,11^.

Generally, data regarding seroconversion after SARS-CoV-2 infection in young children is scarce. The few studies that exist describe seroconversion rates as low as 37.5% one to two months after positive SARS-CoV-2 PCR testing^12^. Most of the studies focusing on this age group assess children in clinical settings^8,12^. Only little data is available for young children in non-clinical community settings^9,13^. Seroconversion rates might also vary between different viral variants^14^, which requires a continuous updating of seroconversion rates throughout the COVID-19 pandemic.

In addition to age, other potential determinants of seroconversion have been identified, such as sex, time interval between symptom onset or a positive PCR testing and antibody testing, severity of disease, and viral load^3–9,12^.

In order to better understand seroconversion in young children, we addressed the following research questions: (a) To what extent do seroconversion rates differ between young children and adults? (b) What factors are associated with seroconversion in young children and adults?

Our results show a significantly higher seroconversion rate in young children than in adults. The analyses depict that seroconversion is associated with viral load (Ct value), time interval between the probable date of infection and antibody testing at follow-up and older age. These results may indicate temporary protection of young children from re-infection or severe disease in the event of re-infection.

## Methods

Data used for this analysis were derived from the COALA study and collected between October 2020 and June 2021, when the SARS-CoV-2 ancestral and alpha-variant were predominant in Germany.

### Study design

Methods of the underlying COALA study are described elsewhere^15^. In brief, the COALA study (“Corona-outbreak-related examinations in daycare centers”) was a prospective, longitudinal study, investigating the transmission dynamics of SARS-CoV-2 in 30 child daycare centers and connected households. Therefore, the selection of participants was related to SARS-CoV-2 outbreaks in daycare centers all over Germany. Infection trajectories of affected children and their contacts were monitored via repeated PCR testing, symptom recording, and capillary blood sampling as well as via a written questionnaire about previous SARS-CoV-2 infections and exposure to SARS-CoV-2-infected individuals. The Ethics Committee of the Berlin Medical Association has reviewed the COALA study and approved the implementation of the study (Eth-39/20). Participation in the study was voluntary and all participants were informed about the objectives and contents of the study as well as data protection and gave their written informed consent. Every participant was assigned to a sequential study number (ANR) in order to ensure pseudonymization of the study documents and material. COALA is registered in the German Clinical Trial Register (DRKS-ID = DRKS00023501).

### Sample characteristics

In the COALA study, 114 individuals tested positive for SARS-CoV-2 via PCR (45 young children 1-6 years, 69 adults). Antibody results were available for 97 participants (38 young children, 59 adults). For 17 participants (seven young children, ten adults) no measurement of antibodies was performed because no appointment for the blood sampling could be made, blood sampling was refused, or because the sample quality of the blood specimen was insufficient. Participants originated from a non-clinical community setting and were asymptomatic or had mild symptoms of COVID-19 (i.e., rhinitis, headache, sore throat, cough, melalgia, and fever, see also ref. ^16^).

### Sampling

To examine SARS-CoV-2 antibodies, capillary blood was taken at two points of time: The first was taken at the beginning of the study period at the first home visit (=baseline sampling), 4 to 6 days after the testing date of the index case. The index case was defined as the first positive case within the daycare group that was diagnosed with SARS-CoV-2 via PCR and reported to the local health authority. The second blood sample (=follow-up sampling) was taken approximately five weeks after the baseline sampling visit according to the study protocol if the participating individual tested positive for SARS-CoV-2 through PCR either prior to the study period by health authorities or in PCR testing during the study period. (see also Supplementary Fig. 1).

### Laboratory analyses

Dried blood samples (DBS) were analyzed for SARS-CoV-2 IgG antibodies using the commercial qualitative antibody assay “Anti-SARS-CoV-2 ELISA (IgG)” (sensitivity 94.4%—blood sample collected >10 days after symptom onset; specificity 99.6%) by the manufacturer Euroimmun (Euroimmun Medizinische Labordiagnostik AG, Lübeck, Germany). The test detects antibodies directed at the spike antigen (S1 antigen). A validation study was performed to verify the applicability of the test for dried blood samples. After evaluation of the results, the ratio threshold for seropositivity provided by the manufacturer (≥1.1) was adapted to ≥0.94^17^. Indeterminate results were considered negative. The test results are semi-quantitative, i.e., they can only be categorized as positive (≥0.94 ratio) or negative (<0.94 ratio) without quantification in terms of titer determination.

The test quality of the PCR measurements was ensured by measuring human c-myc^18^.

### Determination of seroconversion rates

To establish comparability between the seroconversion rates in young children and adults, we determined the probable date of infection for each participant, considering information on exposure and symptom onset (if any), antibody status at first blood draw, and date of first positive PCR test (for further information see ref. ^19^). Due to our study design, the timing of the first positive PCR within the course of the infection varied among study participants. As described in other publications, symptoms and symptom onset are no reliable parameters for the onset of SARS-CoV-2 infections in children^20,21^. Therefore, we decided to determine the individual probable dates of infection as the starting points for analyses of individual infection courses.

To assess a potential association between seroconversion and viral load, we selected the PCR results with the highest viral load (i.e., lowest Ct value; further details see ref. ^22^) of each participant for the analyses.

### Statistics and reproducibility

Characteristics of participants are shown as mean/ median, ±SD (standard deviation) for continuous variables, and as % for categorical variables.

A backward stepwise logistic regression approach was used to identify possible predictors of the outcome seroconversion at testing at follow up. The following variables were taken into consideration: sex, a variable indicating whether the person tested was an adult or a child, viral load (lowest Ct value), presence of symptoms, and the time interval between the probable date of infection and antibody testing at follow-up.

Regardless of the criterion used to determine which variables to exclude, viral load (lowest Ct value), the time interval between the probable date of infection and antibody testing at follow-up, and a variable indicating the person tested being an adult were selected for further analysis, while sex and symptoms were dismissed.

The final model presented in the text was re-run with Firth bias correction^23^. This correction was used to account for the small sample size in some categories (only one seronegative case in the group of young children). This small sample size also is the reason for presenting results as odds ratios, based on a logistic regression model, and not as relative risks, since the odds ratio estimate is less subject to small sample bias than the risk ratio estimate.

In addition, the multivariable logistic regression analysis was corrected for the unequal distribution of antibody-positive individuals in young children and adults. For this purpose, the indicator variable indicating whether the tested person is an adult was inserted.

Analyses were performed using STATA/SE 17 (StataCorp LLC, College Station TX, USA), as well as R version 4.1.2 (2021-11-01) “Bird Hippie” Copyright (C) 2021 for stepwise backward logistic regression package ‘MASS’ version 7.3-58.3 and for regression analysis with Firth bias correction package ‘logistf’ version 1.24.1.

### Reporting summary

Further information on research design is available in the Nature Portfolio Reporting Summary linked to this article.

## Results

### Participant characteristics

Data from 91 participants (38 young children, 53 adults) was included in the analyses. Six participants had to be excluded due to detectable antibodies at the baseline sampling: for four participants, the questionnaire data indicated that these persons were vaccinated against COVID-19. For the other two participants, no information was given in the questionnaire about a previous infection. We assume that these participants had an undetected SARS-CoV-2 infection in the past.

The basic characteristics of the study population (total) and the seropositive subgroup are described in Table 1.

**Table 1:** Participant characteristics—total (*n* = 91) and seropositive (*n* = 72).

Characteristics	Participants					
	Children			Adults		
	Total (*n* = 38)	Serostatus		Total (*n* = 53)	Serostatus	
		Sero-positive (*n* = 37)	Sero-negative (*n* = 1)		Sero-positive (*n* = 35)	Sero-negative (*n* = 18)
Sex, female, *n* (%)	18 (47.4)	17 (45.9)	1 (100)	31 (58.5)	19 (54.2)	12 (66,7)
Age, median, years, ±SD (min–max)	5, ±1.4 (1-6)	5, ±1.4 (1-6)	3	37, ±8.7 (18-56)	37, ±8.4 (22-56)	37.5, ±9,4 (18-56)
SARS-CoV-2 infection status, *n* (%)						
PCR positive in COALA	35 (92.1)	35 (94.59)	0 (0)	51 (96.2)	35 (100)	16 (88.9)
PCR positive determined by health authorities^a^	3 (7.9)	2 (5.41)	1 (100)	2 (3.8)	0 (0)	2 (11.1)
Viral load (lowest Ct value), mean Ct, ±SD (min–max)^a^	26.1, ±4.8 (16-34.9)	26.1, ±4.8 (16-34.9)	0^a^	24.8, ±5.5 (14.9-37.2)	23.9, ±4.9 (14.9-33.2)	26.6 ± 6.2 (16.58-37.2)
Symptoms, *n* (%)	27 (71.1)	26 (70.3)	1 (100)	50 (94.3)	34 (97.1)	16 (88.9)
Time interval between the probable date of infection and antibody testing at follow-up, mean, days, ±SD (min–max)	49.6, ±3.4 (45-56)	49.5, ±3.3 (45-56)	54	48.1, ±4.3 (42-55)	48.7, ±4.4 (43-55)	46.5 ± 3.8 (42-55)

^a^No Ct values were available for PCR tests from health authorities; ±SD standard deviation; years.

More than 90% of both young children and adults had a positive PCR test during the study period. Five cases were reported as positive by national health authorities using official PCR testing, but possessed negative PCR test results during study sampling afterwards.

In those cases, no Ct values were available. Viral load and interval between the probable date of infection and antibody testing at follow-up were comparable between adults and young children. Symptoms were more frequent in adults than in young children, both in the entire cohort and in the seropositive subgroup.

### Seroconversion findings

The seroconversion rate in young children was significantly higher than in adults: 97.4% (*n* = 37) of the young children and 66% (*n* = 35) of the adults had detectable S-antigen IgG antibodies at the follow-up sampling about seven weeks after the probable date of infection (*p* < 0.001).

### Multivariable logistic regression analysis for association between seroconversion and several determinants

For 86 of 91 study participants, regression analysis was performed (Table 2). Five cases had to be excluded due to missing Ct values. Seroconversion was associated with viral load (Ct value), time interval between the probable date of infection and antibody testing at follow-up, and age (seroconverted person being an adult). The higher the viral load (i.e., a lower Ct value) and the longer the time interval between the probable date of infection and antibody testing, the more likely it was for participants to show seroconversion. The indicator variable for age (the person tested being an adult) showed that participants aged over 18 years were less likely to seroconvert.

**Table 2:** Multivariable logistic regression analysis for association between seroconversion and several determinants (*n* = 86).

	Odds ratio (95% CI)
Viral load (lowest Ct value)	0.84 (0.70–0.97)
Time interval between the probable date of infection and antibody testing at follow-up	1.27 (1.06–1.62)
Person tested being an adult	0.03 (0.0002–0.23)

*CI* confidence interval.

## Discussion

Ninety-seven percent of young children, versus 66% of adults, had developed SARS-CoV-2 antibodies at day 43-56 after the date of probable infection. The difference in seroconversion rates between young children and adults was statistically significant. Independent of age, a high viral load at the time of acute infection was positively associated with seroconversion. A longer time interval between the probable date of infection and antibody testing at follow-up increased the odds of seroconversion, although the variability in time span within the entire study group was small (up to 14 days). That was to be expected, however, as we sampled all participants according to study protocol at similar times.

Interestingly, other studies found a considerably lower seroconversion rate among young children, compared to our results. Whereas 97% of young children in our study had developed SARS-CoV-2 antibodies, Toh et al. detected antibodies in only 37%^9^, and Ruedas-Lopez et al. in 37.5%^12^ of tested children. One explanation for the substantial differences to our study results may be the level of viral load. Young children in our study population had relatively high viral loads (median Ct value of 26). In accordance with our results, Toh et al. concluded from a subgroup of their study sample that high viral loads (Ct < 26) may be associated with higher seroconversion rates^9^. Also, Walker et al. found that participants (≥16 years) with higher viral load (Ct < 25) showed seroconversion significantly more frequently than participants with lower viral load (Ct > 25)^24^.

A positive association between high viral load and seroconversion, regardless of age, was also shown in our analysis. We hypothesize that young children were more likely to have developed antibodies following a SARS-CoV-2 infection than adults due to their fast and strong immune system^20,25^.

Our findings on seroconversion in adults (66%) are comparable with seroconversion rates also assessed by S-antigen antibodies against SARS-CoV-2 reported in literature ranging from 57.7% to 76.6% to 90%^9,26,27^.

With respect to the time interval between the probable date of infection and antibody testing at follow-up, we can assume that we have captured the seroconversion of all individuals. As studies have shown that IgG antibodies against spike protein are reliably detectable in blood samples after more than 14 days and up to 4 to 5 months after the first positive PCR or symptom onset^3,4,6,7,28,29^.

Strengths of our study are the assessment of young children in a non-clinical, community setting, the prospective study design including the collection of biological samples (repeated PCR tests, antibody testing at two points of time), and close monitoring of symptoms.

Nevertheless, some limitations should be considered. Our study has a relatively small sample size that limits statistical power. However, comparable studies have similarly small numbers of participants, presumably due to the young age of the study subjects^8,9,12^. Although participants were selected in the context of SARS-CoV-2 outbreaks in daycare centers all over Germany, the sample of daycare centers was not randomized and thus may not be representative. Overall, 59% of the invited young children and adults of a daycare group and their close contacts participated. Reasons for non-participation were not evaluated.

Furthermore, the results of the indicator variable suggest that young children seroconvert more frequently than adults. However, the number of cases in our study is too small to estimate this precisely. Therefore, we do not discuss age as an associating factor for seroconversion.

Concerning the laboratory testing of antibodies, the results are semi-quantitative. Since the aim of the study was to investigate seroconversion, not the strength of the immune response, we did not perform quantitative measurements of the antibody concentrations. Overall, it is possible that under-reporting of infected individuals may have occurred due to false-negative PCR samples. Prolonged unrefrigerated transport in hot temperatures can lead to false-negative PCR results. However, the majority of our samples were collected in winter and spring. We, therefore, consider under-reporting, in the sense of an increased number of false-negative samples, to be very unlikely. In addition, all samples arrived at the laboratory within the time frame specified by the study.

Viral variants of SARS-CoV-2 may induce different immunological responses. Our findings are derived from the ancestral and alpha SARS-CoV-2 variants that were predominant at the time of data collection in 2020/2021 in Germany. The results may therefore be not transferable to subsequent variants. Recent analyses in Australian children after infection with Delta- or Omicron SARS-CoV-2 variants show that seroconversion rates in young children (6 months-17 years; median 3–4 years of age) one month after a positive PCR were 100% for the Delta variant (*n* = 35/35 children) and 81% for the Omicron variant (*n* = 13/16 children)^14^. These results are compatible with ours but differ from previously reported results for this age group.

Our findings contribute to the overall picture of published but still scarce data on seroconversion after infection with SARS-CoV-2 in young children.

### Supplementary information

Supplementary Information

Reporting Summary

## Data Availability

The datasets presented in this article are not readily available because the authors confirm that some access restrictions apply to the data underlying the findings. The dataset cannot be made publicly available because informed consent from study participants did not cover public deposition of data. However, the source dataset underlying the findings is archived in the “Health Monitoring” Research Data Centre at the Robert Koch Institute (RKI) and can be accessed by researchers on reasonable request. On-site access to the dataset is possible at the Secure Data Center of the RKI’s “Health Monitoring” Research Data Center. Requests to access the datasets should be directed to “Health Monitoring” Research Data Center, Robert Koch Institute, Berlin, Germany (fdz@rki.de).
