# Supplementary material for: SARS-CoV-2 seroconversion in children attending daycare versus adults in Germany between October 2020 and June 2021
Source: Commun Med (Lond). 2023 Sep 15;3:124. doi: 10.1038/s43856-023-00352-3 (PMC10504330; doi:10.1038/s43856-023-00352-3)
Supplement: Supplementary file 1 — Supplementary Information [file 43856_2023_352_MOESM1_ESM.pdf]

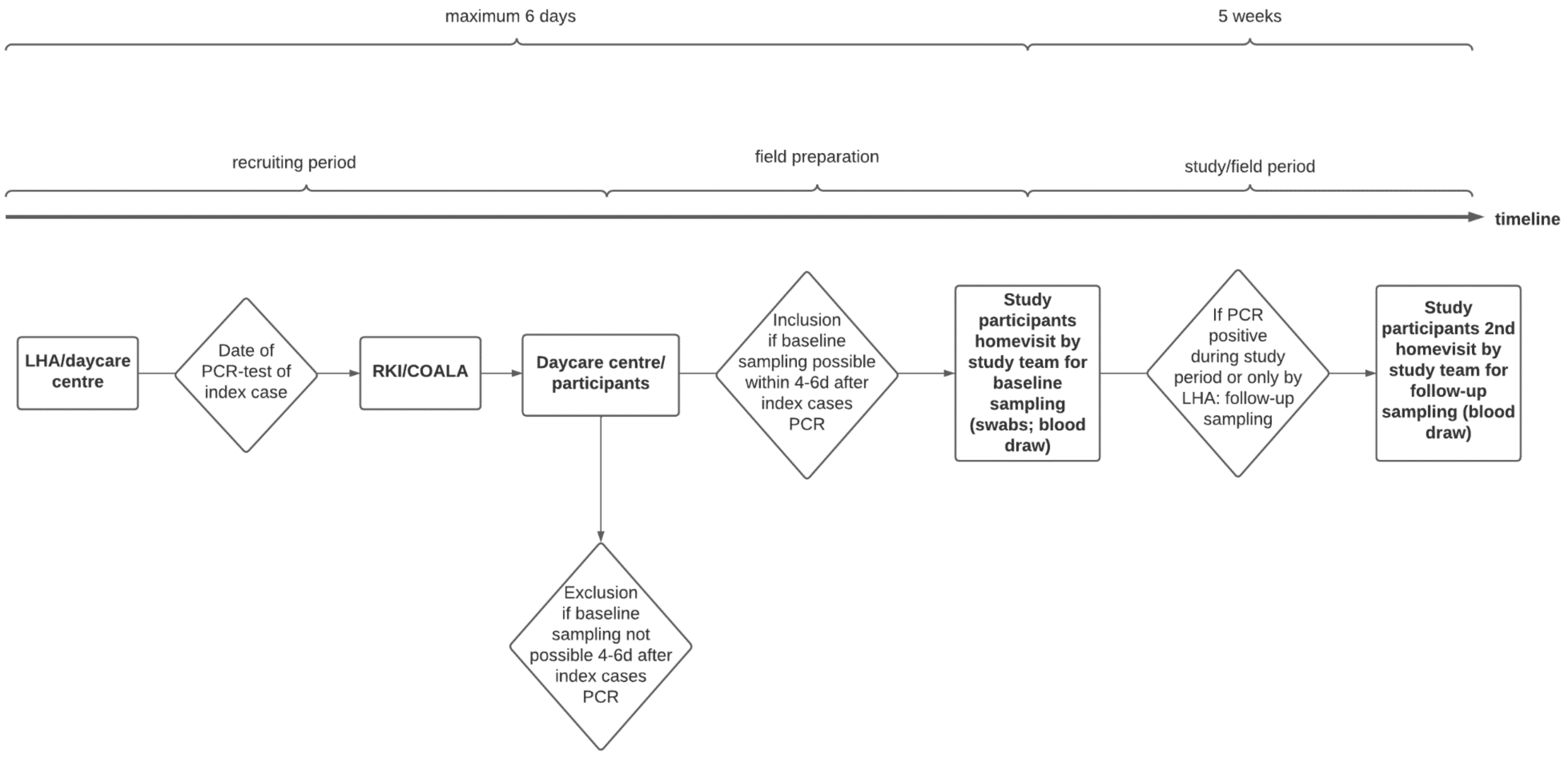

Supplementary Figure 1 Recruitment and inclusion process of COALA study participants: In 2020, 3.4 million children in Germany were cared for in about 52.870 daycare centres. The study presented here (COALA) included a total 943 participants (282 daycare-children, 139 children and 522 adults) from 30 daycare centres throughout Germany (https://doi.org/10.1007/s00103-021-03449-z; DOI: 10.3278/9783763973279 p. 26). (LHA: local health authority; RKI: Robert Koch Institute; COALA: "Corona outbreak-related examinations in daycare centres"; PCR: polymerase chain reaction; d: days)
